# Baseline assessments of research capacity, capability and culture in UK local authorities: reflections from evaluators embedded in Health Determinants Research Collaborations

**DOI:** 10.1186/s12961-025-01323-x

**Published:** 2025-05-26

**Authors:** Lauren Bell, Rachel Chapman, Charlotte Ashton, Claire Batey, Jack Brazier, Elizabeth Castle, Arundeep Chaggar, Julian Elston, Faye Esat, Hannah Goldwyn Simpkins, Leonard Ho, Cath Quinn, Jessica Sheringham, Demelza Smeeth, Irene Stylianou, Simon Twite, James Woodall, Beck Taylor

**Affiliations:** 1 Health Determinants Research Collaboration Coventry, Coventry City Council, Coventry, CV1 2GN United Kingdom; 2 University Hospitals Coventry and Warwickshire NHS Trust and Coventry City Council, Coventry, United Kingdom; 3 Health Determinants Research Collaboration Islington, Islington Council, N1 1XR London, United Kingdom; 4 Health Determinants Research Collaboration Newcastle, Newcastle City Council, Newcastle upon Tyne, NE1 8QH United Kingdom; 5 Health Determinants Research Collaboration Somerset, Somerset Council, Somerset, TA1 4DY United Kingdom; 6 Health Determinants Research Collaboration Lambeth, London Borough of Lambeth, London, SW2 9QQ United Kingdom; 7 Community and Primary Care Research Group (CPCRG), University of Plymouth, Plymouth, PL6 8BX United Kingdom; 8 Health Determinants Research Collaboration Doncaster, City of Doncaster Council, Doncaster, DN1 3BU United Kingdom; 9 Health Determinants Research Collaboration Tower Hamlets, London Borough of Tower Hamlets, 160 Whitechapel Road, London, E1 1BJ United Kingdom; 10 Health Determinants Research Collaboration Aberdeen, Aberdeen City Council, Aberdeen, AB10 1AB United Kingdom; 11 University College London, London, WC1E 6HB United Kingdom; 12 Health Determinants Research Collaboration Lambeth, Public Health, London Borough of Lambeth, London, SW2 9QQ United Kingdom; 13 Health Determinants Research Collaboration Wakefield, Leeds Beckett University, Leeds, LS1 3HE United Kingdom; 14 University of Warwick and Honorary Consultant in Public Health, NHS England, London, United Kingdom; 15 Plymouth Health Determinants Research Collaboration, Plymouth City Council, Plymouth, PL1 3BJ United Kingdom; 16 MRC Unit for Lifelong Health and Ageing at UCL, Department of Population Science & Experimental Medicine, Institute of Cardiovascular Science, Faculty of Population Health Sciences, UCL, London, United Kingdom

**Keywords:** Evaluation, Collaboration, Inequalities, Research capacity, Local government, Social determinants of health

## Abstract

**Background:**

In the United Kingdom, local government is well placed to conduct and apply research regarding the wider determinants of health. However, local authorities often lack sufficient research infrastructure to support research capacity, capability and culture. Since 2022, the UK National Institute for Health and Care Research has funded 30 Health Determinants Research Collaborations (HDRCs) to develop this infrastructure. HDRCs are hosted by local authorities collaborating with universities and other partners to strengthen a culture of evidence-informed decision-making. HDRCs are conducting local evaluations, including baseline assessments of local authority research capacity, capability and culture.

**Methods:**

A national peer-support group was formed to support shared learning amongst teams evaluating HDRCs. Here, as embedded evaluators from 10 HDRCs, we present reflections on the planning, delivery and interpretation of baseline assessments. Reflections were gathered via group discussions and written submissions. All 10 HDRC baseline assessments explored local authority research capacity, capability and culture, and two also studied early HDRC team collaboration.

**Results:**

Competing priorities during early HDRC implementation called for pragmatic and timely baseline assessment methods. Most HDRCs developed baseline surveys, though interviews and focus groups were conducted by some. Despite similar aims, methods varied substantially according to local contexts. Evaluators often adapted existing validated survey tools, for example, from health settings, as none were identified for use across local government. Definitions of research also ranged from academic definitions to broader notions of evidence. Useful insights were gathered across diverse samples to aid implementation locally, however, low response rates were received to all-staff surveys and heterogeneous approaches limited comparison across HDRCs. Findings contributed to recommendations for evaluating and developing HDRC activities (e.g. communications and training provisions) appropriate for local authorities with stretched resources. Where measured, collaborations were functioning well, with recommendations to enhance communication.

**Conclusions:**

The early contexts and challenges of HDRCs influenced pragmatic baseline assessments. Methods were often chosen to capture baseline contexts rapidly, and they will be refined and complemented by additional evaluation methods as HDRCs progress. Developing new validated measures and an agreed definition of research for local authorities may strengthen understanding of research capacity, capability and culture across local government.

## Background

Local government plays a critical role in the lives and health of its residents. In the United Kingdom (UK), local authorities have responsibility for a range of services and policies that impact the wider determinants of health and health inequalities [1, 2], including housing, transport, education, employment, culture, regeneration, environments, public health and social care [3]. Local government often also engages in innovation to support local development, yet operates in political and financially challenging contexts [4]. Increasingly, local authorities are tasked with making critical decisions about how to prioritize limited financial resources [5]. To ensure that local authority decisions are underpinned by the best available research evidence and meet the needs of local communities [6], an enabling research culture and infrastructure is required [7]. This research infrastructure refers to research-related resources, facilities, funding, expertise, professional development pathways and data and governance systems that can support the production of research and mobilization of knowledge into practice [8].

A well-established research infrastructure facilitates high-quality health research in UK research institutions [9, 10]. The National Institute for Health and Care Research (NIHR), the largest funder of UK health research, allocates around £1.3 billion annually to generate health and care research, enabling significant advances in knowledge and health outcomes [11]. NIHR, along with other health research funders, provide funding for research infrastructure, as well as for research studies and programmes. Although UK local authorities are uniquely well placed to build evidence about the wider determinants of health, they have historically been allocated significantly less funding for research programmes and infrastructure than health settings [12].

Funders have recently sought to better understand and increase research resources available to local government, including NIHR-commissioned studies part of the Local Authority Research Systems (LARS) work [13, 14]. The evidence base has reported that existing research infrastructure is often inaccessible to local authorities, with limited staff time and research training [14–17]. Barriers to collaboration between local authorities, universities and communities have included discordant research timelines and distrust [7, 14, 16]. The Local Authority Champions of Research (LACoR) study, funded by the Health Foundation, also found a lack of consensus across local government about what counts as evidence, and that despite interest in using evidence, capacity pressures and governance issues restricted capacity and capability for research [18]. Recommendations to increase research co-production with local government included increasing academic researchers’ understanding of local government, facilitating communication and knowledge brokering spaces and engaging with council leaders to ensure political relevance [19].

Since 2022, NIHR has awarded funding to establish 30 UK Health Determinants Research Collaborations (HDRCs), each initially for 5 years. This funding is to build research capacity, capability and culture for evidence-based decision-making on the health determinants. HDRCs are hosted by local authorities with universities, voluntary and community organizations and other local organizations acting as partners usually within a local geographical area [20]. Each HDRC intends to develop research infrastructure suitable for local contexts, with diverse collaborators and approaches. Yet, all HDRCs share similar aims to build local authority research infrastructure, promote a culture of always using research evidence in decision-making, involve local people and communities and ultimately improve health determinants and reduce inequalities [20]. Typical workstreams to develop research capacity and capability include training and skills provisions, data processes, governance infrastructure, embedded researcher models and strengthening involvement of communities and other partners in research.

There is extensive evidence regarding healthcare and applied health research collaborations, including NIHR-funded infrastructure programmes [21]. In particular, evidence from NIHR-funded Collaborations for Leadership in Applied Health Research and Care (CLAHRCs) suggested the important influence of local contexts in shaping delivery of the programmes, such as according to the local leaders’ vision, yet there was a notable lack of sharing from those delivering CLAHRCs about their practical learning and experience [21]. HDRCs are the first NIHR collaborations hosted in local government. Evaluating the first examples is therefore crucial, and there is likely to be significant value in sharing learning and experiences from those embedded in the implementation. An independent study of the national HDRC programme is underway, and HDRCs are each conducting evaluations to uncover the influences of local contexts, inform local developments and generate transferable evidence. In 2023, individuals planning local HDRC evaluations established a national peer-support group, meeting monthly to share learning and resources. This group highlighted the unique challenges to evaluating research collaborations in local authority contexts, and we (the authors) produced this reflective article to share our learning.

### Article aims

The purpose of this article is to describe our early experiences, methods and preliminary insights from baseline assessments of a national programme of local authority-hosted research collaborations (HDRCs) to influence policy, practice and evaluation methodology in similar contexts.

## Methods

### Involvement of HDRCs in this article

Evaluation teams from 10 HDRCs contributed to this article (Aberdeen, Coventry, Doncaster, Islington, Lambeth, Newcastle, Plymouth, Somerset, Tower Hamlets and Wakefield). HDRCs launched in 2022 (phase 1), except Somerset and Wakefield, which launched in 2023 (phase 2), and Islington, which began a “development year” in 2022 and launched in 2023 [20, 22].

Evaluators in the peer-support group with a finalized “baseline assessment” protocol by June 2024 were invited to contribute to this reflective article. “Baseline assessment” refers to the initial phase of data collection in HDRC evaluations. The first year of HDRCs involved foundational activities such as recruiting staff (including evaluation teams), setting programme aims and objectives and developing evaluation protocols [22]. Baseline assessments therefore may not have preceded all HDRC implementation, but still did reflect the early phases of implementation within the first 2 years of funding. HDRCs were not required to contribute findings to this article, reflecting the value of HDRCs sharing their experiences and learning from developing baseline assessments to date.

### Approach to developing this article

Evaluation teams’ decisions and reflections about baseline assessments were gathered during group discussions and written submissions. Two online meetings were held and recorded in June and September 2024 to produce detailed notes; formal transcriptions were not obtained. Each HDRC submitted written information about the design, development and conduct of their baseline assessment, plus findings and reflections to date. HDRC Coventry (L.B., R.C., B.T.) collated and interpreted these contributions, highlighting areas for further discussion in the second meeting. The article was drafted (L.B., R.C., B.T.) and then revised by all authors.

## Results

### Reflections and experiences of planning and delivering HDRC baseline assessments

Each HDRC evaluation team’s baseline assessment is described in Table 1.

**Table 1:** Descriptions of baseline assessments conducted by HDRCs contributing to this article

	Phase 1 HDRCs								Phase 2 HDRCs	
	Aberdeen	Coventry	Doncaster	Islington	Lambeth	Newcastle	Plymouth	Tower Hamlets	Somerset	Wakefield
Evaluation team	Embedded evaluation by HDRC Skills & Culture workstream. Mixed team with roles in local authority	Embedded evaluation led by HDRC evaluation workstream. Mixed team with roles in local authority, NHS and university	Embedded evaluation with HDRC evaluation lead researcher working in the local authority	Embedded evaluation with HDRC evaluation leads working in the local authority and university	Embedded evaluation by HDRC team in the local authority and students’ input	Embedded evaluation led by HDRC knowledge mobilization lead working in the local authority	Embedded evaluation led by university researchers in the HDRC team	Embedded evaluation team in the local authority with advice from university partners. Also plans for external evaluation contract	Embedded evaluation by HDRC team, led by business development and research intelligence workstream, and supported by other HDRC workstreams, embedded researchers from partner university and voluntary, community, faith and social enterprise (VCFSE) sector	Embedded evaluation led by researchers in the HDRC’s university partner
Baseline assessment aims	Understand different aspects of local authority research culture and capacity	Understand (1) different aspects of local authority research culture and capacity (2) how well the HDRC are functioning together	Understand different aspects of local authority research culture and training preferences	Obtain baseline measures to enable evaluation of the process and outcomes of HDRC	Understand different aspects of local authority research culture and capacity	Understand different aspects of local authority research culture and infrastructure, and identify staff interested in being involved in research activity	Understand different aspects of local authority research culture and capacity	Understand (1) different aspects of local authority research culture and capacity (2) how well the HDRC collaboration are functioning together	Understand different aspects of research culture and capacity in the local authority and VCFSE. Identify research training needs and priority areas	Understand different aspects of local authority research culture and capacity
Ethics and governance approvals	Local approvals	Formal university ethical approval and local approvals	Formal university ethical approval and local approvals	Formal university ethical approval and local approvals	Local approvals by HDRC leads	Approval sought from HDRC director and service manager and Asst Chief Executive’s directorate leadership team	Local approvals	(1) Local approvals from local authority directors (2) Formal university ethical approval and local approvals	Formal university ethical approval and compliance with local governance processes	Formal university ethical approval and local approvals
Design	Online survey – Quantitative questions plus open-ended questions	Online survey for each of evaluation aims one and two. Both surveys included quantitative measures plus open-ended questions	Online survey	(1) Facilitated team or group surveys with local authority and voluntary sector groups, (2) Interviews with staff and partners and (3) Online survey	Online survey – Quantitative measures plus open-ended questions	Online survey – Quantitative measures plus open-ended questions. The survey was complemented by individual interviews in one council directorate	Online survey – Quantitative measures plus open-ended questions	(1 + 2) Online survey – Quantitative measures plus open-ended questions	Focus groups, interviews and online surveys with local authority and VCFSE representatives	Interviews with purposefully sampled local authority participants
Public and professional involvement and piloting	Developed by evaluation team only. Piloted with HDRC and local authority team	Two HDRC public co-applicants involved in tool choice; input from HDRC collaborators on plans. Piloted in local authority HDRC team	Involvement of steering group with academic, community group and local authority representatives. Piloted in two local authorities	Resident involved in developing approach and methods; input from HDRC embedded researchers. Piloted in local authority teams	Developed by evaluation team only. Piloted with HDRC and local authority team	Input from local authority colleagues. Piloted in local authority teams	Input from HDRC embedded team. Piloted with local authority team	(1) Input from local authority colleagues (2) Input from HDRC collaborators on proposals. Piloted in local authority teams	Consultation with local authority professionals about best engaging participant across the organization. Local authority colleagues were also involved in developing the topic guide	Developed by evaluation team only
Inclusion or adaptation of existing tools and frameworks	Bespoke 32-question tool informed by RCC but developed to suit local authority contexts	(1) RCC and perceived value subscale of SEER, language adapted for clarity in local authority contexts (2) Wilder Collaboration Factors Inventory, language adapted to HDRC	Research and Development Culture Index adapted to local authority terminology	(1) Shortened version of SEER to reduce completion time and duplication, language adapted to a group (2) Informed by CFIR (3) Adapted Research Culture and Capacity Tool	Adapted RCC and SEER, changes to improve relevance and ease to participants and reduce completion time	Six bespoke questions developed for local authority contexts, though informed by RCC and SEER domains	Bespoke questions informed by research capacity and capability domains in existing tools developed in social care, adapted [23–25] to enhance relevance to local authority	(1) Local Authority Research System (LARS) framework (2) Nuffield Partnership Assessment Tool	Bespoke questions informed by previous work locally	Topic guides informed by previous work locally and evidence-informed typology [17]
Participant eligibility	All council staff and councillors	(1) All council staff councillors (2) HDRC members	All council staff and councillors	All council staff and councillors and voluntary sector partners	All council staff and councillors	All council staff and councillors	All council staff and councillors	(1) All council staff and councillors (2) HDRC members	All council staff, VCFSE organization representatives	Council service directors and leaders
Recruitment	Promoted via council intranet and service managers	(1) Broad promotion across council channels and all-staff email. Targeted promotion to leadership roles. completion (2) Direct email, with reminders	Broad promotion via all staff email, available on intranet, word of mouth	Broad promotion across council channels, via HDRC strategic delivery group and manager briefings	Broad promotion across council channels and all-staff email, shared through professional networks and engagement events	Broad promotion across council channels and all-staff email cascaded by directorate business managers	Broad promotion across council channels and all-staff email. Engaged with council leaders to promote	(1) Broad promotion across council channels and all-staff email (2) Email to eligible participants, with three reminders	Targeted recruitment via email and joining serve-wide team meetings. Supported by local authority ambassadors. VCFSE organizations were recruited via email and local authority and voluntary alliance communication channels	Direct email to eligible participants
Data collection dates	June 2023–October 2023	December 2023–February 2024	September 2023–December 2023	June 2024–February 2025	March 2024–June 2024	January 2024–March 2024	October 2023–July 2024	(1) October–December 2021 (2) February 2024	September 2024–January 2025	June 2024 November 2024

*HDRC* Health Determinants Research Collaboration, *RCC* research culture and capacity tool, *SEER* Seeking, Engaging with and Evaluating Research, *CFIR* Consolidated Framework for Implementation Research, *VCFSE* voluntary, community, faith, and social enterprise sector

### Evaluation teams

Contributing evaluators to this article were embedded members of a HDRC and involved in wider HDRC meetings or workstreams. Evaluation teams were composed of either local authority professionals, academic researchers or individuals from a mix of collaborating organizations, and were diverse in seniority, research and practice expertise. Our degree of embeddedness also varied. Some worked full time across different HDRC activities, and others delivered the evaluation part time alongside roles outside the HDRC. Being embedded offered us valuable insights, for example, to support plans to align with each local authority’s norms and processes, and accounting for contextual factors present at each locality. Our embeddedness also led to challenges balancing capacity for evaluation work alongside labour-intensive HDRC implementation, leading to more pragmatic baseline assessment designs. As evaluators embedded in our respective HDRCs, we also considered the role of subjectivity in evaluation planning and delivery. To mitigate this potential limitation, some HDRCs invited critical peer-review of evaluation protocols from other HDRCs and/or had involved wider collaborators in shaping aspects such as templates for qualitative framework analysis. These approaches would need to be developed in future phases of evaluations so that the benefits of being embedded researchers were maximized.

### Governance processes

In total, 6 out of the 10 HDRCs obtained formal ethical approval from a university committee for the baseline assessment. Approval was obtained where mandatory for university-employed evaluators and/or to facilitate journal publication. Remaining HDRCs determined that ethical approval was not required for this low-risk evaluation activity, though followed local processes (e.g. communication team or director approval). Academic publication was often not a primary goal. Instead, evaluators needed to move quickly to capture timely baseline assessment, with the university ethics process sometimes perceived as a barrier. Some evaluators described challenges navigating usual local authority practice (no ethical approval due to lack of infrastructure) and university processes (mandatory for any data collection). Regardless, ethical practices such as informed consent and anonymous participation were adopted. For some HDRCs, baseline assessments also provided an opportunity to scope and test local HDRC research governance processes being developed.

### Aims and methods of HDRCs’ baseline assessments

#### Aims and purpose

The conduct of HDRC baseline assessments served numerous purposes. Importantly, baseline assessments intended to record early contexts and variables upon which data collected in later phases of the HDRC could be compared. In other words, assessments were to enable longitudinal data collection towards a summative evaluation of each HDRC’s progress and impact throughout the funding period. Many HDRCs had logic models or theories of change for how visions would be realized, and evaluation plans also provided an opportunity to test and refine these theories. Baseline assessments were also carried out to gather local insights that could formatively influence HDRC activities in real time (e.g. to inform and refine training opportunities and communication plans and align developments to council priorities). In addition, it was expected that exposure to the baseline assessment would be the first introduction to the HDRC for many prospective participants, and assessments therefore were also viewed as a tool to raise awareness and engagement with the HDRC.

HDRCs’ stated aims for their baseline assessment varied, but all aimed to understand their local authority’s research capacity, capability and culture at a very early phase of the HDRC. Tower Hamlets investigated this in 2021, prior to the HDRC, which served as a baseline assessment of research activity in the local authority at the time. Other HDRCs collected baseline assessment data during the first year or two of the HDRC funding period. The topics explored varied, though included involvement with and perceptions of research, training and qualifications, knowledge and skills, organizational support for research and familiarity with research infrastructure. HDRCs Islington and Somerset also explored these topics with local voluntary, community, faith and social enterprise collaborators.

HDRCs Coventry [28] and Tower Hamlets also aimed to understand how the HDRC team leading the implementation were functioning and collaborating at this early phase. Aspects including shared vision, leadership, involvement, communication and clarity of roles and plans were explored to provide recommendations and monitor collaboration over time.

#### Baseline assessment design

The design of the baseline assessment was decided by individual HDRCs and influenced by local contexts, academic expertise, published evidence and the team’s previous experiences. As evaluators, we reflected that competing demands around early HDRC implementation meant in-depth academic research design was often balanced with available resource. As a result, most HDRCs developed surveys to capture baseline insights, despite longer-term plans for more comprehensive mixed-methods evaluations.

***Surveys***. Nine HDRCs developed online surveys hosted on Microsoft Forms, Qualtrics, Google Forms or Survey Monkey. Surveys included quantitative scales or items, supplemented with open-ended questions to gain qualitative insight. Lengths varied from brief “snapshot” surveys (e.g. six items) to in-depth 20-min surveys (e.g. approximately 80 items). These differences reflected some HDRCs favouring shorter surveys to increase recruitment and others prioritizing longer surveys with validated tools, seeking to enable reliable testing and comparison across studies. All HDRCs intended to repeat surveys (e.g. (bi)-annually), though anticipated modifying tools or recruitment approaches to reflect changing contexts and incorporate learning.

***Qualitative and mixed-methods approaches***. HDRC Wakefield used a wholly qualitative approach using interviews and focus groups to gather in-depth information and identify thematic areas, building upon previous qualitative work conducted locally. HDRCs Islington, Somerset and Newcastle planned mixed-methods baseline assessments involving surveys and interviews or focus groups to triangulate evidence. In HDRC Somerset’s baseline assessment, interviews and focus groups were the prioritized methods, though the topic guide was developed into a survey to collect data from participants who did not wish to participate in the qualitative work. HDRC Islington devised a topic guide informed by the Consolidated Framework for Implementation Research (CFIR) [29]. CFIR was also used by HDRC Coventry to analyse qualitative responses to the team collaboration survey.

Amongst the 10 HDRCs involved in this article, qualitative methods were prioritized by the phase 2 HDRCs, whereas phase 1 HDRCs more typically adopted survey methods. Phase 2 HDRCs shared that they had observed some of the recruitment and other challenges observed with surveys, and alongside intentions to build on previous work conducted locally, had prioritized qualitative methods to further explore the nuances related to the research question. Given the low number of phase 2 HDRCs included in this article, however, this pattern may not reflect the approaches taken by the wider network of 30 HDRCs.

In addition, several HDRCs reported plans for additional mixed and qualitative work as part of their longer-term evaluation plans; for example, plans to conduct interviews or focus groups towards the end of HDRC year two. In this way, it was important to recognize that baseline assessments were only the initial phase of broader mixed-methods evaluations planned by HDRCs throughout the funding period.

### Survey content

#### Defining research

Definitions of “research” provided to participants differed across HDRCs. Some aligned with the academic research-focussed UK Health Research Authority definition, whereby research is considered an attempt to derive generalizable or transferable knowledge using scientifically sound methods [30]. HDRCs using this definition described how they selected it to enable assessment of change in academic research activity likely to be supported by major funding bodies and to lead to high-impact research outputs. Other HDRCs favoured broader definitions of “research” activity, including routine data analysis, consulting, auditing or quality improvement and benchmarking exercises. This more inclusive definition, which differs from the Health Research Authority definition, intended to recognize and value the different components of “research” activity and “evidence” used in local authority and community contexts and support participants to consider how evidence relates to their roles.

#### Use and adaptation of existing tools and frameworks

Independently, and within our peer-support group, we searched for, shared and critiqued validated tools for measuring research culture and capacity. A formal academic systematic review was not undertaken, though HDRCs each carried out database searches and shared and discussed tools in the peer-support group. No tool validated for use across UK local government contexts was identified. Tools most commonly considered were the research culture and capacity (RCC) tool [26] and Seeking, Engaging with and Evaluating Research (SEER) [27]. No HDRC had used a validated tool verbatim, and there were varying degrees of adaptation. HDRC Coventry [28] used RCC with minor modifications (items specified “academic” research). HDRC Doncaster used the Research and Culture Development Index [31], and adapted language from the original healthcare context to suit local government. HDRC Aberdeen modelled survey questions on the ADKAR (Awareness, Desire, Knowledge, Ability and Reinforcement) change framework [32] already trusted and familiar to staff and councillors in their local authority. HDRC Plymouth’s survey was informed by previous work and tools to investigate research culture in adult social care [23–25]. Tower Hamlets’ pre-HDRC baseline survey was a modified tool initially delivered as part of NIHR funded Local Authority Research System (LARS) work in Bradford [33]. Several HDRCs adapted existing tools via partial inclusion of subscales, removing items, or using the tools to create new questions. HDRC Newcastle mapped the underlying domains in RCC and SEER and devised one question per domain to create a shortened tool.

The approach of many HDRCs to adapt and modify existing tools was because of predicted issues with applying the original validated tools to local authority contexts. In particular, tools were considered too long to engage busy participants working in the local authority. Tools also included technical and scientific language (e.g. “systematic reviews”) that assumed previous exposure to research, where such language may disengage participants (from both the survey and the HDRC) or impede validity. We therefore agreed that developing new tools to investigate local government research capacity, capability and culture would be beneficial to future developments to better facilitate reproducibility. HDRCs anticipated that subsequent repetitions of the survey would be modified to incorporate baseline learning and the changing contexts of HDRCs. In doing do, evaluators will need to consider a balance between retaining questions that can enable comparison with the baseline timepoint, as well as incorporating supplementary questions to capture the changing contexts and developments.

HDRCs measuring collaboration functioning adopted either the Wilder Collaboration Factors Inventory Index [34] (Coventry) or the Nuffield Partnership Assessment Tool [35] (Tower Hamlets). Minor wording changes to both were made to increase relevance, as again tools developed for contexts similar to HDRCs were not found.

#### Additional survey questions

HDRCs created additional questions to investigate aims, including about participants’ previous research-related training and/or training preferences, involvement in research or awareness of research infrastructure. Most HDRCs collected professional characteristics such as role and department. Some collected personal characteristics such as education, age, gender and ethnicity. Characteristics were intended to help understand survey reach and generalizability, and where sample sizes permitted, compare groups.

### Professional and public involvement

Most HDRCs involved professionals in planning evaluations, most often wider HDRC or local authority colleagues known to the HDRC, for example, public health colleagues where HDRCs were co-located within public health teams. This approach resulted from the challenges experienced, at this baseline timepoint, with explaining the complexities and contexts of HDRCs. In other words, it was complex to involve wider collaborators (i.e. those not known to the HDRC) in the methods design when the contextual understanding of HDRCs had not yet developed. In future phases of the evaluation, awareness and communications around the HDRCs are expected to be more advanced. This may enable wider involvement and further development of appropriate and insightful evaluation methods. In the development of baseline assessments, close engagement with local authority staff increased suitability of methods and was expected to enhance ownership for implementing recommendations. Piloting led to changes to reduce survey length, reframe questions and amend participant consent processes. Involving multiple collaborators contributed challenges when diverse perspectives created unwieldy surveys, which was anticipated to reduce completion rates.

Three HDRCs involved members of the public in developing plans, recruitment approaches or tool choice, which improved appropriateness of methods. Members of the public were already involved in HDRC work, for example, as grant co-applicants, and were typically familiar with the contexts and ambitions of HDRCs. Whilst public involvement is an important cornerstone of HDRCs, payment processes and recruitment pathways were also being established alongside baseline assessment work, limiting the scale of public involvement in the work at this time.

### Participants and recruitment

Across HDRCs, all local authority staff, regardless of role or team, plus councillors were eligible to participate in baseline surveys. Some HDRCs prioritized strategies to recruit staff with decision-making responsibilities where research was considered more relevant.

Survey recruitment strategies included all-staff emails, internal communications, adverts in offices, word of mouth, embedding in email signatures and promotion at events and meetings. Direct engagement with senior managers aimed to encourage participation and/or top-down dissemination, and additional methods were employed to target certain groups, for example, inclusion in councillor bulletins. Face-to-face promotion, where staff were offered refreshments whilst participating on tablet devices, reportedly substantially improved uptake in HDRC Lambeth. Conversely, there was a report that proactive recruitment of certain groups (e.g. councillors) was avoided due to local contexts, and some HDRCs reported groups (e.g. staff without work email addresses) that did not receive survey invitations directly. We reflected on the importance of understanding and responding to local contexts. For example, some local authorities had higher office attendance where surveys could be more effectively promoted in person. In other local authorities, remote working was more prevalent, and online group chat channels were more suitable for survey promotion. Surveys also needed to align and be visible alongside other important staff surveys and communications. Decisions were frequently made to maintain parity with other council surveys by not offering incentives (e.g. vouchers or competition entries). Other strategies were recruiting via trusted gatekeepers and enabling anonymous participation.

Recruiting to qualitative methods, HDRC Wakefield adopted maximum-variation purposeful sampling to interview key individuals (e.g. service directors). Ensuring genuine benefit to prospective participants was also recognized as important when recruiting to focus groups. In future phases of recruitment, the findings and changes made as a result of baseline work must be clearly communicated to encourage further participation.

In the two HDRCs using additional surveys to measure the quality of the collaboration, eligible participants were HDRC colleagues involved in implementation or with a specific HDRC role. These participants were recruited via email with reminders and promotion in meetings.

### Findings from HDRC baseline assessments

At the time of writing, seven phase 1 HDRCs (Aberdeen, Coventry, Doncaster, Lambeth, Newcastle, Plymouth, Tower Hamlets) had baseline findings available reporting local authority research capacity, capability and culture (Table 2). HDRCs Coventry and Tower Hamlets additionally contributed findings from surveys assessing early collaboration functioning (Table 3). HDRCs’ baseline assessment data were collected between June 2023 and July 2024 for a period between 1 month and 5 months (with the exception of the pre-HDRC work conducted in Tower Hamlets, which collected data October to December 2021). Key findings are summarized below.

**Table 2:** Findings and recommendations related to local authority research capacity and culture measured by five HDRCs

Results	Aberdeen	Coventry	Doncaster	Lambeth	Newcastle	Plymouth	Tower Hamlets
Sample size	*N* = 268. Responses from 3.1% of council staff and 28.9% of councillors	Relevant data from *N* > 250, including 101 complete responses, reflecting approximately 2–4% of council staff	*N* = 58, 2% of council staff	*N* = 282 responses, including 225 complete responses, reflecting approximately 10% of council staff	*N* = 100 complete responses, approximately 3% of council staff with email addresses	*N* = 60 2–3% of council staff	*N* = 142 responses, approximately 3% of council staff
Data collection	June–October 2023	December 2023–February 2024	September–December 2023	March–June 2024	January–March 2024	October 2023–July 2024	October–December 2021
Participant characteristics	Around one quarter of participants were teachers, and otherwise mainly from City Growth (11%) and Children’s and Family Services (10%)	More than 20 responses were received from 8 (of 13 total) council directorates (< 20 responses from 5 directorates); 72% had higher education qualification, and more than one quarter were managers or leaders	More than 10 responses were received from 3 service areas 45% had previously undertaken research, which was largely as a result of a higher education qualification; 31% were managers	More than 20 responses from 5 (of 8) different directorates; 77% had at least a higher education degree	More than 10 responses were received from 6 (of 7 total) directorates; 85% had higher education and 27% were managers	Responses were from all 5 directorates (15 subdirectorates), including 18 strategic/senior leaders (30%); 59% had least a higher education degree	Participants were from Place (26% of participants), Resources (25%), Children and Culture (20%), Health (18%) and Governance (11%)
Findings related to research culture, capacity or infrastructure	Most had been involved in research when making decisions (78%), writing reports (72.8%) and accessing and drawing upon internal data sources (71.3%). Respondents tended to use and apply existing research rather than conduct primary studies Around half agreed that the council’s strategic direction and policies are based on evidence. Less than one third thought that the council works closely with partner organizations in research projects	One quarter had been involved in at least one research-related activity in the past 12 months, most often attending conferences. One quarter had applied research evidence to their work. Perceptions of research as moderately valuable in a council context Low–medium scores were reported for aspects of organizational research culture and capacity. Results indicated limited awareness of existing research infrastructure, and limited resources (time, capacity) for teams to deliver research	Organizational research culture differed between service areas, with public health scoring the highest on the validated research culture index and the highest levels of research activity Teams with high levels of measurable service delivery reported the lowest research activity or knowledge of research evidence being applied to their work, but described an appetite to learn and use research	A total of 48% reported research-related activities as being part of their role, and 38% were frequently involved in data collection activities. The least frequent research activity was securing research funding A total of 46% searched for evidence reviews when developing policies A notable barrier to research involvement was having allocated time for research	One quarter had tools needed to engage in research, which could indicate existing skills are underutilized. Capacity was a barrier to research involvement. Support to be involved in research varied depending on person’s manager and location in organization Lack of awareness of existing research, or where to go for research Self-selecting respondents were interested in, and valued, research, and most thought senior leaders supported research, though were unsure about how research translated into policy/practice Qualitative findings indicated that there was inconsistency in how research was accessed and carried out across similar roles in the council	A total of 37% had formal training in research, 63% thought research was extremely/very relevant to their role, 68% had the opportunity to discuss research and 68% accessed research literature, with 73% to accessing non-council resources Promoting evidence-based practice to guide service developments, supported by senior manager were the strongest areas research practice/culture and organizational infrastructure and planning less developed. Although 67% reported research would improve the quality of their work, only 50% reported wanting to be more involved in research, and only 48% wanting to take part in research skills training (particularly analytical skills), with only 23% reporting the capacity to do so. Time and capacity were reported as the largest barriers to undertaking more research	A total of 21% of respondents frequent used research findings, predominantly in-house research and national statistics, and 48% sometimes do and would like to do so more. Main barriers – not knowing how to access or where from. At organization level – 48% agreed council values use of research evidence for decision-making, 33% felt that senior leaders encouraged use of evidence; 30% of respondents reported that their department or service had commissioned research, predominantly from private sector (80%), with only 12% and 8% from the voluntary and community and higher education sectors, respectively
Findings related to research-related training, knowledge and skills	More than half reported research skills (e.g. searching literature and collecting data); 11.2% had research qualifications and 19% had professional research experience. The most frequent research related activity was training or continued professional development (80.2%)	Participants had varied levels of research training and skills. There were some examples of higher-level training or professional development. Training most often occurred prior to the participant’s role in the council	More than 75% of respondents were interested in future training and development opportunities to develop research skills. Findings indicated topics for training should centre around: effective impact and evaluations and how to apply evidence into practice and co-production with communities. For training and development opportunities there was an equal split between those that preferred face to face engagement and those that preferred online	For 70%, the primary motivator for engaging in research was skill development, though more resources to support training were required. Findings indicated topics for training should be around applying for and securing funding and offering workshops or modules to help develop knowledge	Qualitative findings indicated some staff reported existing research capabilities that were not always fully utilized	In relation to skills and confidence across a range of research activities there was generally a fairly even distribution between extremely and no skills or confidence across a wide range of research areas, with staff reporting being more skills and confidence in engaging user and presenting findings and less in planning research, recruitment and protecting participants from harm	A total of 80% reported that they would like to use research evidence more in their role. Respondents were asked what support would be most helpful. Formal training seen as least helpful. Regular bulletins on research relevant subjects (56%), informal opportunities to meet council colleagues to share expertise (16%), in-house training on how to use research (11%), informal in-house opportunities to find out more on using research in my work (11%), access to formal external training (6%)
Key recommendations from findings	Consider separate, bespoke training for councillors and staff including hands-on training with internal (particularly for councillors) and external data sources, and training on how to better use research Enable increased involvement of councillors and staff in external research activities Consider influence of time pressures and work demands when planning activities	Respond to diversity in existing skills and experience through a training offer suitable for introductory to advanced levels Develop research governance to support the local authority to lead and carry out research Raise awareness of the HDRC and other external research infrastructure Recognize and respond to challenging contexts and time pressures when planning activities	Develop a variety of bespoke training and development opportunities including face-to-face and online workshops. Develop a community of research practice and case studies across several service areas. Create space to share and discuss practice, barriers and methodologies Create infrastructure to carry out co-produced research	Enhance research training and support for research-related skills and activities where confidence lower Consider strategies to enhance time capacity for staff involvement in research Create space to share and discuss research initiatives and resources Raise awareness of HDRC work	Develop community of practice, communications and capacity building opportunities Develop communication and awareness raising activities Recognize the existing diversity of research skill and experience that was showcased. Harness what is already there as well as build skills	Explore work with key council teams, senior leaders and management, to create processes and infrastructure that support organizational research and learning. Work “bottom up”, involving council staff, academics and community partners early in research development to promote relevant and impactful research. Facilitate increased access to evidence, including peer-reviewed journal articles, and increase capacity and capability to assess quality of research. Provide introductory training around research to build staff knowledge, skills and confidence, initially around refining research questions, planning research, recruiting participants and ensuring safe participation in research. Support staff career development including through fellowships and research funding opportunities. Review staff research ideas and consider how those aligning with HDRC priorities can be best supported	Address “quick wins” – only 10% could access peer reviewed journal articles online, 20% not using research evidence do not as they do not know where to access it – provide comms and support Develop/deliver internal programme of training. Connect with NIHR (looking into identifying learning needs in non-NHS settings) Explore how to strengthen the championing by senior leaders of using and doing research across the organization Take opportunities to strengthen our engagement with higher education and voluntary and community organizations when doing and commissioning research

*HDRC* Health Determinants Research Collaboration,* N* number, *NIHR* National Institute for Health and Care Research

**Table 3:** Findings and recommendations related to collaboration functioning measured by two HDRCs

	Coventry	Tower Hamlets
Sample sizes	*N* = 20 (53% of eligible HDRC members)	*N* = 20 (65% of eligible HDRC members)
Data collection	December 2023–February 2024	February 2024
Participant characteristics	Working in local authority (*n* = 7), university (*n* = 5), NHS (*n* = 3), voluntary, community or social enterprise organization (*n* = 3) and/or other (including public contributor) (*n* = 3)	Working in local authority (*n* = 9), university (*n* = 7), voluntary, community organization (*n* = 2) and/or other (including public contributor) (*n* = 2)
Findings related to collaboration functioning	On a five-point agreeability scale, the three items with highest satisfaction were mutual respect, understanding and trust (*M* = 4.30); members see HDRC as being in their self-interest (*M* = 4.35); and unique purpose (*M* = 4.38). Lowest scores (reflecting neutral/no opinion) were for multiple layers of participation (*M* = 3.15); sufficient funds, staff, materials and time (*M* = 3.20); and development of clear rules and policy guidelines (*M* = 3.35). Due to the substantial scale of the HDRC and challenging contexts, qualitative comments suggested greater reflective discussion was needed, as well as identifying ways to enhance capacity to engage amongst collaborating organizations	The partnership was largely achieving its aims and objectives and the principles of partnership performing reasonably well. The highest performing partnership principle was develop clarity and realism of purpose (17.7 / 24), whilst the lowest performing partnership principle was recognize and accept the need for partnership (16.7 / 24). Findings indicated the partnership had clear values and success criteria. However, the vision could be inconsistent between partners and successes should be better communicated. Clarifying when and how partners should work more independently, and ensuring the partnership does not become too council-dominated, were indicated
Key recommendations from the findings	Collaboratively review internal communication pathways to enable reflective practice Identify ways of increasing capacity and connectedness of collaborators, including via streamlined communication, prioritization of available resource and engaging widely with individuals from across collaborating organizations	Ensure the HDRC vision is clear for all partners. Consider strategic operational arrangements to support autonomy and workload distribution, and that partners all value involvement Consider mechanisms for how successes are communicated

*HDRC* Health Determinants Research Collaboration, *N* number, *M* mean

### Findings from baseline local authority research capacity, capability and culture surveys

#### Survey participants

The number of participants in each HDRC ranged from 58 to 282, with response rates between 2% and 3% of local authority employees, with the exception of Lambeth at 10% (Table 2). Participants worked across departments, although some groups (e.g. teachers, public health officers) were overrepresented. HDRC Aberdeen received responses from 28.9% of councillors, whilst others received far fewer responses from this group. HDRCs with longer surveys identified high drop-out rates, though final response rates were consistent with shorter surveys. We also collectively reflected about likely sampling bias. Relative to the wider local authority workforce, participants were often more likely to be in managerial roles and/or have higher educational qualifications, which may be linked with greater research experience (i.e. via university degrees). We also considered that surveys about research would recruit respondents more interested in research, and therefore research capacity and capability may have been overestimated (a positive bias).

#### Survey results and interpretations

Across HDRCs, survey participants reported diverse levels of research-related skills and experience (Table 2). Some groups, such as public health teams, reported higher levels of research involvement, aligning with preliminary qualitative findings by HDRC Wakefield. Due to heterogeneity in survey tools and definitions of research, we could not make direct comparisons between HDRCs. However, findings indicated varying levels of research capacity and capability. Almost half of participants in one local authority reported research activities being part of their role, whereas in another one quarter were involved in any research-related activity in the previous year. Overall, more participants reported applying evidence to decision-making than reported involvement in primary research. Barriers to using, doing and leading research included lack of infrastructure (e.g. limited journal access and research governance processes) and insufficient time and resource. Many participants wanted to learn more about research, however, and some participants reported that existing skills and training were underused in their local authority roles.

Findings provided useful insights into participants’ preferences and needs, for example, for training on applying for research funding, and a desire to connect with professional researchers.

Responding to the challenging demands on resource and capacity within local authorities will be fundamental to implementing functional and sustainable HDRCs. Key recommendations for HDRCs and local authorities, amongst other groups, are summarized in Table 4.

**Table 4:** List of key recommendations organized by target group

Evaluators
• Coordinate with peer-evaluators in similar contexts to act as critical friends • Consider recording and sharing experiential learning of the process • Maximize the opportunities of being embedded evaluators, including through implementing recommendations and change • Ensure time and opportunity to involve collaborators, including the public, in developing and piloting plans and methods, ensuring plans align with local priorities • Embed and refine multiple methods approaches, particularly where early contexts may demand pragmatic methods initially • Engage diverse, proactive and inclusive recruitment strategies to reach across local government teams
Health Determinants Research Collaborations (HDRCs) and similar settings
• Clearly embed plans for formative and summative evaluation, including a programme theory or theory of change • Engage with evaluation findings to influence the development of plans and implementation of infrastructure, training and other opportunities suitable for local authorities with limited resource and capacity • Prioritize effective collaboration, communication pathways and clear responsibilities for collaborators implementing complex collaborations such as HDRCs
Local authorities
• Promote staff participation in evaluations to support comprehensive understanding of the contexts and the development of appropriate and effective interventions • Co-develop evaluations and tools to ensure that methods are suitable for individual local government contexts and support recommendations to be actioned
Funders
• Consider that research outputs valuable to HDRCs or local government may not be published journal outputs • Consider how local evaluations are resourced given the important influence of local contexts and formative insights • Consider the influential and complex contexts of local government and how then research may be accessed, applied, and conducted
Further research
• Co-develop an agreed definition of research for local government contexts, recognizing that “research” can occupy different meanings and definitions • Co-develop validated tools to measure research culture, capacity and collaboration that are widely tested across local government

*HDRC* Health Determinants Research Collaboration

### Findings from baseline assessments of collaboration functioning

#### Participants

HDRCs Coventry and Tower Hamlets each recruited 20 participants from their HDRC teams, with response rates of 53% and 65%, respectively (Table 3). Fewer responses were from HDRC members more peripherally involved, for example, steering or executive committee members, compared with workstream leads.

#### Results and interpretation

Results from both HDRCs indicated that their collaboration was functioning well, though different survey tools used by each HDRC explored different domains of collaboration (Table 3). For example, Coventry participants were highly satisfied with respect, understanding and trust in the collaboration, and reported that the HDRC had a unique purpose aligned with individual interests [28]. In Tower Hamlets’ survey, participants appraised their HDRC as achieving its aims to date, with clear values and success criteria. Scores and qualitative comments in both HDRCs resulted in recommendations to revise communication pathways, including strategies to facilitate more reflection as a collaboration, clarify roles and share successes to build on the collaborative foundations established. Key recommendations for HDRCs are summarized in Table 4.

## Discussion

In this article, embedded evaluators from 10 HDRCs shared reflections regarding the planning, delivery and interpretation of baseline assessments. Through involvement in a peer-support group, as evaluators we benefitted from shared critical discussions about baseline assessment methodology, strategies, tools and the definitions and framing of concepts including “research”. Fellow evaluators acted as critical friends, and the forum provided an opportunity to share knowledge and expertise in a group with diverse academic and local authority backgrounds. Despite exploring opportunities to align data collection methods, local contexts contributed to HDRCs adopting different approaches. There was variation in team and public involvement, ethical and governance approvals, methodology, definitions of research, use and adaptation of validated tools and participant recruitment. In summary, the peer-support group provided a valuable opportunity to share and critique plans to evaluate a complex programme, yet evaluators had to prioritize methods that were appropriate for local contexts, and thus a cohesive methodology across HDRCs was not determined. This article has resulted in a number of key recommendations for evaluators, HDRCs, local authorities and funders, as well as suggestions for further developments in research (Table 4).

All 10 HDRC baseline assessments aimed to assess local authority research capacity, capability and culture, and two also explored HDRC team culture. Most HDRCs used surveys, though survey design varied without suitable “off the shelf” tools available and the influence of local contexts. Local authority surveys also experienced low response rates, and evaluators recognized the competing political and financial challenges of many local authorities that may have contributed to this outcome [36]. However, findings provided valuable insights to inform the development of HDRCs and learning that can be taken forward in evaluations of HDRCs and similar contexts.

### Considerations for evaluating research collaborations hosted in local government

Given the challenges of early HDRC implementation, including staff recruitment and competing priorities [22] and unavoidable pressures in local authorities, as embedded evaluators we frequently developed pragmatic baseline methods to capture timely assessments. The baseline assessments provided early insights to pragmatically influence HDRC development and evaluation, and many teams were planning additional work to evaluate other aspects of HDRC implementation and impact moving forwards. To date, HDRCs had applied baseline findings to HDRC implementation in a number of ways. These included using the survey as a mechanism to identify potential research champions, identifying tailored approaches to engage and support different council teams and identifying the need for specific infrastructure development around data governance and storage. More commonly across HDRCs, the findings informed HDRC training offers with diverse components to reflect the varying levels of baseline research capacity across individuals and teams. A key strength of evaluators being embedded in HDRCs was that such findings could be actively mobilized within HDRCs in timely ways.

Given the novel contexts of local authority-hosted research collaborations, involvement of collaborators was anticipated to be highly valuable [37], and was reported to have shaped appropriate evaluation methods that could maximize recruitment, response rates, engagement and ownership of recommendations from the findings. The breadth of involvement at this baseline timepoint, however, was recognized to be largely limited to local authority and HDRC professionals in most HDRCs. Wider involvement was restricted where processes to recruit and reimburse members of the public had not yet been finalized and due to the challenges and time needed to support individuals to understand the complex contexts and objectives of HDRCs. In future work and phases, consideration must be given for how to involve wider groups, including embedding necessary timeframes, so that the value of diverse input can be further optimized.

As existing research culture and capacity measurement tools were not validated across local government contexts [26, 27, 31], most HDRCs chose to modify tools or create new ones. Evaluators also defined research differently, from more academic definitions to broader notions of research and evidence, reflecting variation in HDRC workplans and wider debates [38, 39]. These variations limited opportunity to compare findings and highlight how “research” has a different meaning across sectors, which may influence how HDRCs work in practice and how their success will be judged. Though not available at the time of these baseline assessments, work is currently ongoing to develop a new validated tool, and to recognize and define the components of “research” in local government contexts [39]. These developments, if used widely and consistently, may facilitate a more integrated understanding of local government research culture and capacity and support practical application of evidence.

Amongst HDRCs who were able to share findings from baseline assessments, low response rates and likely sampling bias were consistent challenges, for both shorter- and longer-length surveys. A recent review of response rates for surveys with local government professionals reported a highly variable range between 1.4% and 96.7%, with a downward trend in recent decades [40]. Defining the study population is an important consideration when assessing response rates. Response rates amongst HDRC baseline assessments typically used the entire local authority workforce as denominator, yet some groups (e.g. those with “offline” roles and without regular access to a work email) were unlikely to see the survey promotion. The low response rates might also reflect that those in certain roles (e.g. without decision-making responsibilities) may perceive a survey about research culture to not be relevant to their roles. Our HDRC evaluation peer-support group identified feasible and successful recruitment strategies (proactive, visible and regular engagement with council teams) along with others which were less suitable in council contexts (e.g. use of financial incentives). Evidence also supports engagement with collaborators and senior staff who are well placed to endorse surveys and implement findings [41], alongside increased notifications and completion reminders [42].

### Considerations for implementing research collaborations hosted in local government

Baseline assessments provided useful insights for HDRCs building and implementing research infrastructure, developing inclusive training offers and promoting engagement and awareness of HDRCs. Baseline findings from the seven HDRCs with results available suggest that improvements to research infrastructure are required to facilitate local authority involvement with research. Consistent with previous studies, barriers to involvement included insufficient training opportunities, time and capacity [14, 16, 33, 43], and HDRCs may consider ensuring training opportunities and research involvement can effectively align with existing work pressures and activities. Findings from the UK social care workforce have also reported low involvement in research, despite higher levels of interest [23], consistent with findings from HDRCs and wider literature [33, 43]. Indications that existing staff research skills and experience may be underutilized in local government have also been reported [18]. HDRCs are therefore likely to benefit from actively identifying and engaging employees with existing research-related interest and skills to champion the purpose and activities of the HDRC.

Effective collaboration is essential to realize visions such as HDRCs [7]. Two HDRCs measured team culture using different tools, and both reported favourable levels of collaboration at this early phase. Their recommendations aligned with insights gained from another whole-systems programme (ActEarly) that aimed to develop research capacity between local authorities, researchers and communities [44]. The ActEarly evaluation similarly highlighted the importance of clear communication, aims and scope, united vision and resources across partners, flexibility in approach and sufficient levels of resource and infrastructure, whilst recognizing the challenges implementing complex programmes amidst changing and complex contexts [44].

### Strengths and limitations

This article brings together the methods, findings and reflections from 10 UK HDRC baseline assessments, collated in September 2024. It offers a snapshot of baseline contexts and findings from this new, innovative research infrastructure. A practical strength of bringing together learning across HDRC baseline assessments is that individual HDRCs have access to broader, generalizable insights about considerations and challenges for implementing and evaluating HDRCs. As embedded evaluators and insiders in our respective HDRCs, we are well placed to support action on the findings of the baseline assessments. A consideration of this work is that 20 HDRCs are being implemented elsewhere, and their evaluation perspectives, methods, local contexts and points of learning may differ.

## Conclusions

HDRCs are an exciting development in UK research infrastructure, aiming to address the historical gap in funding and research to address the wider determinants of health in local authority and community settings. This work highlights the substantial efforts and achievements of local HDRC evaluation teams, along with the challenges in evaluating research collaborations in complex contexts. It emphasizes the need for tailored evaluation approaches and the development of new tools to avoid duplication and heterogeneity and to enable assessment and comparison of impact across settings. The evidence and insights presented in this article will support ongoing evaluations of HDRCs and similar programmes to develop methodology, deliver rigorous evaluations and generate meaningful evidence of impact.

## Data Availability

No datasets were generated or analysed during the current study.

## Abbreviations

HDRC: Health Determinants Research Collaborations
NIHR: National Institute for Health and Care Research
RCC: Research culture and capacity tool
SEER: Seeking, engaging with and evaluating research tool
UK: United Kingdom
CFIR: Consolidated framework for implementation science
NHS: National Health Service
VCFSE: Voluntary, community, faith and social enterprise sector
